# The Association Between Plasma Copper Concentration and Prevalence of Diabetes in Chinese Adults With Hypertension

**DOI:** 10.3389/fpubh.2022.888219

**Published:** 2022-06-03

**Authors:** Zhixin Cui, Hong Chen, Wenhai Lu, Ping Wang, Ziyi Zhou, Nan Zhang, Zhuo Wang, Tengfei Lin, Yun Song, Lishun Liu, Xiao Huang, Ping Chen, Genfu Tang, Juan Gao, Yong Duan, Binyan Wang, Hao Zhang, Xiping Xu, Yan Yang, Xianhui Qin, Huicui Meng

**Affiliations:** ^1^School of Public Health (Shenzhen), Sun Yat-sen University, Shenzhen, China; ^2^Department of Disease Control and Prevention, Pingdi Center for Public Health Service of Longgang District Shenzhen Municipality, Shenzhen, China; ^3^Graduate School at Shenzhen, Tsinghua University, Shenzhen, China; ^4^Department of Clinical Research, Shenzhen Evergreen Medical Institute, Shenzhen, China; ^5^Department of Cardiology, Peking University First Hospital, Beijing, China; ^6^Key Laboratory of Precision Nutrition and Food Quality, Ministry of Education, Department of Nutrition and Health, College of Food Sciences and Nutritional Engineering, China Agricultural University, Beijing, China; ^7^Shenzhen Institutes of Advanced Technology, Chinese Academy of Sciences, Shenzhen, China; ^8^Institute of Biomedicine, Anhui Medical University, Hefei, China; ^9^Department of Cardiology, The Second Affiliated Hospital of Nanchang University, Nanchang, China; ^10^College of Pharmacy, Jinan University, Guangzhou, China; ^11^School of Heath Administration, Anhui Medical University, Hefei, China; ^12^Department of Neurology, Baoding First Central Hospital, Baoding, China; ^13^Yunnan Key Laboratory of Laboratory Medicine, Kunming, China; ^14^Department of Clinical Laboratory, The First Affiliated Hospital of Kunming Medical University, Kunming, China; ^15^National Clinical Research Center for Kidney Disease, State Key Laboratory for Organ Failure Research, Division of Nephrology, Nanfang Hospital, Southern Medical University, Guangzhou, China; ^16^Guangdong Provincial Key Laboratory of Food, Nutrition and Health, Guangzhou, China; ^17^Guangdong Provincial Engineering Laboratory for Nutrition Translation, Guangzhou, China

**Keywords:** plasma copper, diabetes, adults with hypertension, Chinese, cross-sectional study

## Abstract

**Objective:**

The relationship between plasma copper concentration and prevalence of diabetes in adults with hypertension is unclear. We aimed to determine the association between plasma copper concentration and prevalence of diabetes in Chinese adults with hypertension.

**Methods:**

A total of 2,579 participants (697 cases and 1,882 controls) was included in this cross-sectional study. Plasma copper concentrations were determined by inductively coupled plasma mass spectrometry. Multivariable logistic regression model was used to determine the association between plasma copper concentration and prevalence of diabetes.

**Results:**

According to the logistic regression analyses, the adjusted OR for the prevalence of diabetes in participants with plasma copper concentration ≥109.4 μg/dL was 1.26 (1.00, 1.58) compared with those with plasma copper concentration <109.4 μg/dL (*P* = 0.048). The association was no longer significant following further adjusting for serum high-density lipoprotein cholesterol (HDL-C) concentration as a potential confounder. Stratified analyses demonstrated that serum HDL-C concentration significantly modified the association between plasma copper concentration and prevalence of diabetes (*P-interaction* = 0.043). In the strata of serum HDL-C concentration ≥1.2 mmol/L, a 56% increased prevalence of diabetes was observed in participants with plasma copper concentration ≥109.4 μg/dL compared with those with plasma copper concentration <109.4 μg/dL (*P* = 0.008). No significant relationship between plasma copper concentration and prevalence of diabetes was found in other strata.

**Conclusion:**

Our findings suggested that high plasma copper concentration (≥109.4 μg/dL) was associated with increased prevalence of diabetes in Chinese hypertensive adults with serum HDL-C concentration ≥1.2 mmol/L.

## Introduction

Diabetes has become one of the most important worldwide public health challenges, especially in China (1, 2). The estimated prevalence of diabetes in China significantly increased from 10.9% in 2013 to 12.4% in 2018 (3), and it is estimated that China will be ranked first for the number of adults with diabetes till 2045 (2). Therefore, the identification of risk factors of diabetes is urgently needed for primary prevention of diabetes. Recently, more attention has been given to studying the effects of trace elements on diabetes risk (4–8).

Copper is an essential trace element acting as a catalytic cofactor in some vital enzymes for metabolism and it plays an important role in protein transportation (9, 10). Despite being essential, excessive copper may mediate the formation of excessive damaging reactive oxygen species (ROS) *via* Haber-Weiss and Fenton-like reactions (11), which has been reported as a trigger for insulin resistance (12). The Cu-Superoxide dismutase (SOD) and hydrogen peroxide (H_2_O_2_) system may induce lipid peroxidation (13), which is closely associated with diabetes (14). In addition, excess copper has been reported to reduce the relative abundance of short-chain fatty acid producing bacteria and increase the relative abundance of *Corynebacterium*, which collectively may promote metabolic inflammation and poor glucose control (15). Based on these previous findings, plasma copper concentration, which is frequently used to estimate nutritional status of copper, may be related to prevalence of diabetes (16).

Previous studies investigating the association between plasma copper concentration and prevalence of diabetes are limited and have shown divergent results due to variations in region, sample size and potential confounders adjusted for in analysis models. A meta-analysis (8) has reported that diabetic patients have higher levels of plasma or serum copper than healthy controls. Three case-control studies conducted among Chinese adults are in agreement with the meta-analysis study (17–19), and all of these studies have shown a positive association between plasma or serum copper concentration and diabetes. However, other studies have reported no significant differences in plasma copper levels between diabetic patients and controls (20, 21), or no association between plasma or serum copper and diabetes in European populations (22, 23). These studies were conducted with relatively small sample sizes of Europeans and with a limited number of confounders adjusted for in the models.

Adults with hypertension account for over 40% of adults in China (24). One study considers hypertension and diabetes to be “bad companions”, contributing to many pathophysiological mechanisms underlying cardiometabolic disorders (25). Previous study has also reported that risk of new-onset diabetes in hypertensive individuals is more than twice higher than healthy individuals (26). Therefore, attention should be paid on reducing the prevalence of diabetes in adults with hypertension. In addition, some studies have reported that elevated serum copper concentrations are associated with increased risk of hypertension (27, 28). Due to the lack of study in this topic, it is necessary to assess the association between plasma or serum copper concentration and prevalence of diabetes in adults with hypertension.

This study aimed to determine the association between plasma copper concentration and prevalence of diabetes in a nationwide cohort of Chinese adults with hypertension.

## Materials and Methods

### Study Population

The study population is from an ongoing, multi-centric, community-based, cross-sectional survey initiated in February 2017 in order to identify, register and educate individuals who are at high-risk for both hypertension and elevated total homocysteine [individuals with essential hypertension according to the diagnostic criteria of the 2010 Chinese guidelines for the management of hypertension (29) and elevated total homocysteine (tHcy) (total homocysteine ≥10 μmol/L)] in China. Individuals were openly recruited from various communities according to pre-defined criteria. Exclusion criteria included: severe mental disorders; abnormal laboratory tests or clinical manifestations that render inappropriate participation as evaluated by the investigators; and unwillingness to participate in the study. The study was conducted according to guidelines laid down in the Declaration of Helsinki and was approved by the Ethics Committee of Peking University First Hospital, Beijing, China (Ethics code: 20161231). Written informed consent was obtained from all study participants.

**Figure 1:**
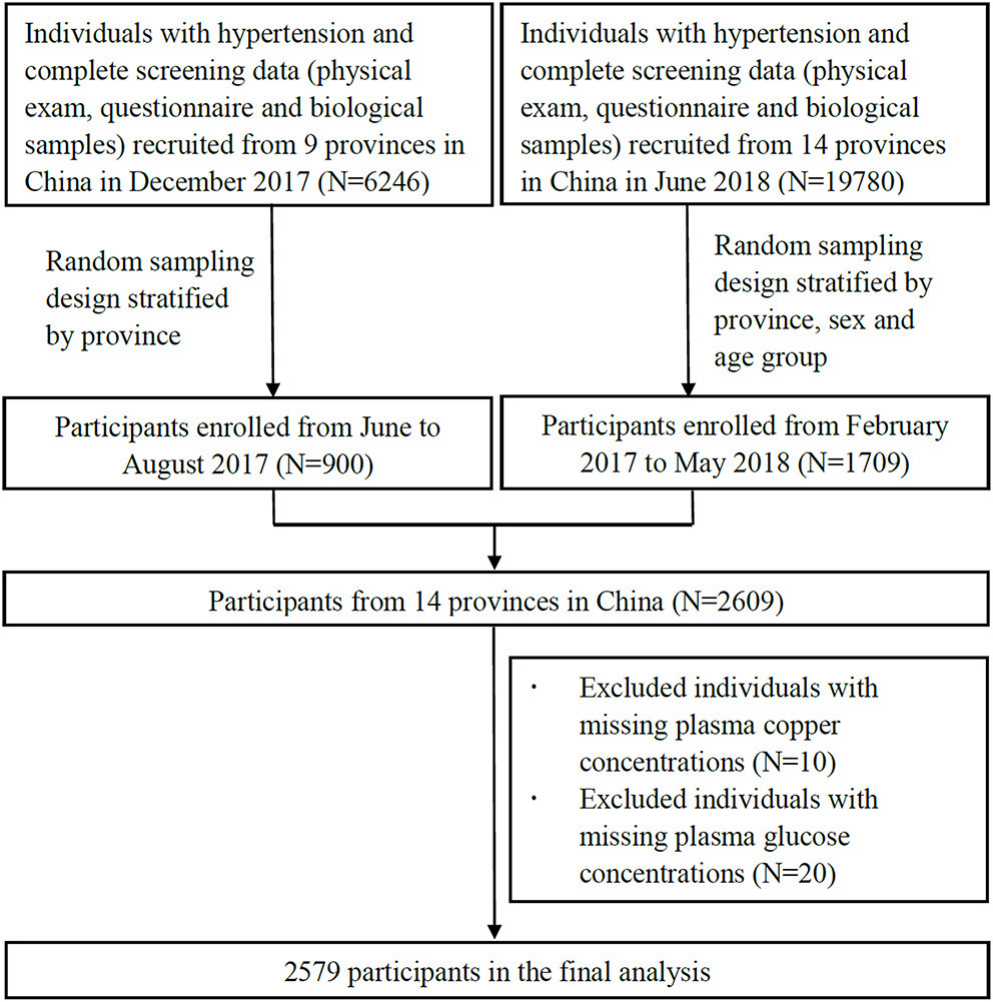
Participant flow chart of the cross-sectional study with 2,579 Chinese adults with hypertension in 14 provinces, China. The nine provinces are Gansu, Liaoning, Beijing, Hebei, Jiangsu, Shanxi, Sichuan, Guangxi and Hunan; the 14 provinces are Gansu, Liaoning, Beijing, Hebei, Jiangsu, Shanxi, Sichuan, Guangxi, Hunan, Heilongjiang, Shandong, Anhui, Ningxia and Yunnan.

Data for the current investigation consisted of two subsamples without duplication extracted by a stratified random sample method from this ongoing study: one from June to August in 2017 and the other from February 2017 to May 2018. In the first subsample, 900 participants were recruited from 9 provinces (Gansu, Liaoning, Beijing, Hebei, Jiangsu, Shanxi, Sichuan, Guangxi and Hunan) by random sampling stratified by province. In the second subsample, 1,709 participants were recruited from 14 provinces (Gansu, Liaoning, Beijing, Hebei, Jiangsu, Shanxi, Sichuan, Guangxi, Hunan, Heilongjiang, Shandong, Anhui, Ningxia and Yunnan) by random sampling stratified by province, sex and age groups. The characteristics of selected and excluded participants were similar in both subsamples (Supplementary Table 1). After excluding those with missing values of plasma copper concentrations (*n* = 10) and plasma glucose concentrations (*n* = 20), a total of 2,579 participants were included in the final analyses (Figure 1).

### Laboratory Assays

Following an overnight fast, venous blood samples were collected from each participant and plasma or serum samples were separated within 30 mins. All samples were stored at −80°C until subsequent laboratory analysis. Plasma copper concentrations were measured by iCAP Q inductively coupled plasma mass spectrometry (ICP-MS; Thermo Fisher, Waltham, the US) in a commercial lab (Beijing DIAN Medical Diagnostics Laboratory, China) as described previously (30). The deviations of measurement accuracy of plasma copper concentrations *via* ICP-MS were between −5.6 and 9.3% for all samples. The detection limit was 10 ng/ml, and the copper concentrations in all plasma samples were above the detection limit in the current study.

Serum fasting glucose, fasting lipids, including total cholesterol (TC), triglycerides (TG) and high-density lipoprotein cholesterol (HDL-C), and tHcy were assessed using automatic clinical analyzers (Beckman Coulter) at the core laboratory of the National Clinical Research Center for Kidney Disease, Nanfang Hospital, Guangzhou, China.

### Assessment of Diabetes

Cases of diabetes were defined as participants with fasting blood glucose ≥7.0 mmol/L (31), a previous physician-diagnosis of diabetes, or who were currently using glucose control drugs from June 2017 to May 2018.

### Assessment of Covariates

Data on demographic and lifestyle characteristics of the study participants, including current smoking, current alcohol drinking, family history of diabetes, use of antihypertensive drugs and use of lipid lowering drugs were collected by the same questionnaire as was previously reported (30). Height was measured to the nearest 0.1 cm and weight was measured to the nearest 0.1 kg. The calculation of body mass index (BMI) was weight in kilograms divided by the square of height in meters. Blood pressure was measured at three consecutive times and the mean values of systolic blood pressure (SBP) and diastolic blood pressure (DBP) were used for analysis.

### Statistical Analysis

R software, version 4.0.1 (Bell Laboratories, NH, USA) was used for all statistical analyses. Plasma copper concentrations and demographic and lifestyle characteristics were presented as mean ± standard deviation (SD) for continuous variables and as proportions for categorical variables. Continuous variables between diabetic cases and controls were compared using *t*-tests and categorical variables were compared using chi-square analyses. Logistic regression models were performed to explore the association between quintiles of plasma copper concentrations (Q1: <80.1, Q2: 80.1 - < 89.4, Q3: 89.4 - < 98.2, Q4: 98.2 - < 109.4 and Q5: ≥109.4 μg/dL) or categories (<109.4 or ≥109.4 μg/dL) and prevalence of diabetes. Model 1 was crude model. Model 2 adjusted for potential confounders, including age (continuous), sex (male or female), current smoking (no or yes), current alcohol drinking (no or yes), BMI (continuous), family history of diabetes (no or yes), SBP (continuous), DBP (continuous), tHcy (continuous), TC (continuous) and TG (continuous). The potential confounders in model 2 were selected with automated stepwise elimination and referring to previous studies. Model 3 additionally adjusted for serum HDL-C concentration (continuous) on the basis of model 2. HDL-C was selected due to the following reasons. Based on our data, participants with serum HDL-C concentration ≥1.2 mmol/L had significantly higher plasma copper concentrations compared to participants with HDL-C <1.2 mmol/L (mean value of 96.6 ± 18.0 μg/dL for HLD-C ≥1.2 mmol/L and 93.9 ≥ 19.7 μg/dL for HDL-C < 1.2 mmol/L; *P* < 0.001 for *t*-test). In addition, there was an inverse association between serum HDL-C concentration and prevalence of diabetes in the current study (Supplementary Table 2). Values of Q1 to Q5 of plasma copper concentration were set as 1–5, respectively, for trend tests. Stratified analysis and potential effect modification was determined for the associations between plasma copper concentration and prevalence of diabetes by age (median, <63.6 years or ≥63.6 years), sex (female or male), current smoking (no or yes), current alcohol drinking (no or yes), BMI (<24.0 or ≥24.0 kg/m2), SBP (median, <141 or ≥141 mm Hg), DBP (median, <87 or ≥87 mm Hg), tHcy (median, <13.9 or ≥13.9 μmol/L), TC (median, <4.2 or ≥4.2 mmol/L), TG (median, <1.4 or ≥1.4 mmol/L) and HDL-C (median, <1.2 or ≥1.2 mmol/L). Likelihood ratio test was used for testing effect modification. Logistic regression models were performed to explore the association between categories of plasma copper concentrations (<109.4 or ≥109.4 μg/dL) and prevalence of diabetes in the stratified analysis. Restricted cubic spline (RCS) was performed to determine the non-linearity potential and dose-response relationship of plasma copper concentration and the prevalence of diabetes. The 10th, 50th and 90th percentiles were selected as the knots, and the plasma copper concentration of 93.66 μg/dL was set as reference value where the estimated OR for prevalence of diabetes was 1. Statistical significance was accepted at a two-tailed *P* < 0.050.

## Results

### Characteristics of Study Participants

A total of 2,579 participants were included in this study (697 cases of diabetes and 1,882 controls). The mean age of total participants was 63.2 ± 13.3 years, 53.5% were males and the mean BMI was 25.0 ± 3.6 kg/m^2^. The mean plasma copper concentration of total participants was 95.3 ± 18.9 μmol/dl (Table 1). Compared with controls, diabetic cases were more likely to be older, have higher BMI, have a family history of diabetes, current use of lipid lowering drugs and have higher TG levels, and more likely to have lower diastolic blood pressure, tHcy, TC and HDL-C levels (*P* < 0.001; Table 1). There were no significant differences in sex, current smoking status, current alcohol drinking status, current use of antihypertensive drugs, systolic blood pressure and plasma copper concentrations between diabetic cases and controls (Table 1).

**Table 1:** Characteristics of 2,579 Chinese adults with hypertension by diabetic status^a^.

**Variables**	**Total**	**Controls**	**Cases**	***P***
*N*	2,579	1,882	697	
Age, *y*	63.2 ± 13.3	62.3 ± 13.7	65.4 ± 11.9	<0.001
Male, *n* (%)	1,381 (53.5)	1,006 (53.5)	375 (53.8)	0.910
BMI, kg/m^2^	25.0 ± 3.6	24.9 ± 3.5	25.5 ± 3.9	<0.001
Current smoking, *n* (%)	502 (19.5)	371 (19.7)	131 (18.8)	0.640
Current alcohol drinking, *n* (%)	458 (17.8)	348 (18.5)	110 (15.8)	0.123
Family history of diabetes, *n* (%)	304 (11.8)	161 (8.6)	143 (20.5)	<0.001
Use of antihypertensive drugs, *n* (%)	1,744 (67.6)	1,282 (68.1)	462 (66.3)	0.403
Use of lipid lowering drugs, *n* (%)	193 (7.5)	123 (6.5)	70 (10.0)	0.003
SBP, mm Hg	142.8 ± 16.9	142.7 ± 17.0	143.0 ± 16.6	0.567
DBP, mm Hg	86.8 ± 11.5	87.4 ± 11.6	85.2 ± 11.1	<0.001
tHcy, μmol/L	17.2 ± 11.8	17.8 ± 12.5	15.8 ± 9.4	<0.001
TC, mmol/L	4.3 ± 1.1	4.4 ± 1.1	4.2 ± 1.1	<0.001
TG, mmol/L	1.6 ± 0.9	1.6 ± 0.9	1.7 ± 1.0	<0.001
HDL-C, mmol/L	1.3 ± 0.3	1.3 ± 0.3	1.2 ± 0.3	<0.001
Plasma copper concentration, μg/dL	95.3 ± 18.9	95.2 ± 18.6	95.4 ± 19.8	0.889

*^a^Data are presented as mean ± SD or n (%)*.

*BMI, body mass index; DBP, diastolic blood pressure; SBP, systolic blood pressure; TC, total cholesterol; TG, triglycerides; tHcy, total homocysteine; HDL-C, high-density lipoprotein cholesterol*.

### Association Between Plasma Copper Concentration and Prevalence of Diabetes

There was no significant association between plasma copper and prevalence of diabetes in model 1, model 2 or model 3 when plasma copper concentrations were categorized into quintiles (all *P for trend* >0.050; Table 2). When Q1 to Q4 of plasma copper concentrations were merged into one group (plasma copper concentration <109.4 μg/dL), a significantly higher prevalence of diabetes was seen in participants with plasma copper concentration ≥109.4 μg/dL compared with those with plasma copper concentration <109.4 μg/dL in model 2 (≥109.4 μg/dL vs. <109.4 μg/dL: adjusted OR = 1.26; 95% CI: 1.00, 1.58; *P* = 0.048; Table 2). The association was no longer statistically significant when HDL-C concentration was further adjusted as a potential confounder in model 3.

**Table 2:** The association between plasma copper concentration and prevalence of diabetes in 2,579 Chinese adults with hypertension^a^.

**Plasma copper concentration, μg/dl**	***N***	**No. of case (%)**	**Model 1**		**Model 2**		**Model 3**	
			***OR* (95% *CI*)**	***P***	***OR* (95% *CI*)**	***P***	***OR* (95% *CI*)**	***P***
**Quintiles**								
Q1 (<80.1)	516	141 (27.3)	Ref		Ref		Ref	
Q2 (80.1–89.4)	516	140 (27.1)	0.99 (0.75, 1.30)	0.944	1.03 (0.77, 1.37)	0.865	1.02 (0.77, 1.36)	0.886
Q3 (89.4–98.2)	515	136 (26.4)	0.95 (0.72, 1.26)	0.740	0.98 (0.73, 1.31)	0.867	0.97 (0.72, 1.30)	0.834
Q4 (98.2–109.4)	516	128 (24.8)	0.88 (0.66, 1.16)	0.357	0.93 (0.69, 1.26)	0.646	0.93 (0.69, 1.25)	0.633
Q5 (≥109.4)	516	152 (29.5)	1.11 (0.85, 1.46)	0.448	1.24 (0.92, 1.66)	0.163	1.20 (0.89, 1.62)	0.231
***P*** **for trend**				0.754		0.332		0.429
Q1–4 (<109.4)	2,063	545 (26.4)	Ref		Ref		Ref	
Q5 (≥109.4)	516	152 (29.5)	1.16 (0.94, 1.44)	0.165	1.26 (1.00, 1.58)	0.048	1.23 (0.97, 1.55)	0.080

*^a^Data are presented as OR (95% CI) estimated by using logistic regression models. Model 1 was crude model. Model 2 adjusted for potential confounders, including age, sex, current smoking, current alcohol drinking, BMI, family history of diabetes, SBP, DBP, tHcy, TC and TG. Model 3 additionally adjusted for HDL-C*.

*BMI, body mass index; DBP, diastolic blood pressure; SBP, systolic blood pressure; TC, total cholesterol; TG, triglycerides; tHcy, total homocysteine*.

### Stratified Analyses on the Association Between Plasma Copper Concentration and Prevalence of Diabetes by Potential Effect Modifiers

As shown in Table 3, serum HDL-C concentration significantly modified the association between plasma copper concentration and prevalence of diabetes (*P-interaction* = 0.043; Table 3). Participants with plasma copper concentration ≥109.4 μg/dL had a 56% increased prevalence of diabetes compared with those with plasma copper concentration <109.4 μg/dL in the strata of serum HDL-C concentration ≥1.2 mmol/L (≥109.4 μg/dL vs. <109.4 μg/dL: adjusted *OR* = 1.56; 95% CI: 1.12, 2.18; *P* = 0.008; Table 3). On the contrary, there was no significant association between plasma copper concentration and prevalence of diabetes in the strata of serum HDL-C concentration <1.2 mmol/L (*P* = 0.884; Table 3). There was no evidence of significant effect modification of the association between plasma copper concentration and prevalence of diabetes by any other potential effect modifiers.

**Table 3:** Stratified analyses and effect modification on the association between plasma copper concentration and prevalence of diabetes in 2,579 Chinese adults with hypertension^a^.

**Variables**	***N***	**Case (%)**	**Plasma copper concentration, μg/dL (mean ±SD)**	***OR* (95% *CI*)**	***P***	***P-interaction***
Age (median), y						0.186
<63.6	1,289	291 (22.6)	93.1 ± 17.3	1.45 (1.01, 2.07)	0.042	
≥63.6	1,290	406 (31.5)	97.5 ± 20.1	1.18 (0.87, 1.59)	0.289	
Sex						0.974
Male	1,381	375 (27.2)	90.8 ± 18.2	1.26 (0.88, 1.80)	0.206	
Female	1,198	322 (26.9)	100.4 ± 18.4	1.27 (0.94, 1.73)	0.121	
Current smoking						0.915
No	2,077	566 (27.3)	95.6 ± 18.4	1.25 (0.97, 1.61)	0.083	
Yes	502	131 (26.1)	94.0 ± 20.7	1.49 (0.84, 2.62)	0.171	
Current alcohol drinking						0.373
No	2,121	587 (28.0)	95.7 ± 19.0	1.18 (0.92, 1.51)	0.182	
Yes	458	110 (24.0)	93.4 ± 18.1	2.05 (1.08, 3.90)	0.029	
BMI, kg/m^2^						0.096
<24.0	983	232 (23.6)	96.9 ± 20.0	1.05 (0.72, 1.52)	0.819	
≥24.0	1,596	465 (29.1)	94.2 ± 18.1	1.44 (1.08, 1.93)	0.014	
SBP (median), mmHg						0.802
<141	1,273	339 (26.6)	94.8 ± 19.1	1.24 (0.89, 1.74)	0.206	
≥141	1,306	358 (27.4)	95.7 ± 18.7	1.30 (0.95, 1.79)	0.101	
DBP (median), mmHg						0.452
<87	1,279	388 (30.3)	96.2 ± 19.8	1.34 (0.99, 1.82)	0.058	
≥87	1,300	309 (23.8)	94.4 ± 17.9	1.16 (0.81, 1.65)	0.420	
tHcy (median), μmol/L						0.849
<13.9	1,285	394 (30.7)	95.1 ± 18.2	1.30 (0.94, 1.78)	0.109	
≥13.9	1,294	303 (23.4)	95.4 ± 19.5	1.25 (0.89, 1.74)	0.201	
TC (median), mmol/L						0.177
<4.2	1,286	397 (30.9)	93.4 ± 19.1	1.03 (0.74, 1.43)	0.868	
≥4.2	1,293	300 (23.2)	97.1 ± 18.5	1.43 (1.04, 1.97)	0.029	
TG (median), mmol/L						0.351
<1.4	1,283	312 (24.3)	96.5 ± 20.0	1.38 (1.01, 1.89)	0.043	
≥1.4	1,252	385 (30.8)	94.0 ± 17.7	1.10 (0.78, 1.54)	0.593	
HDL-C (median), mmol/L						0.043
<1.2	1,288	415 (32.2)	93.9 ± 19.7	0.98 (0.71, 1.35)	0.884	
≥1.2	1,291	282 (21.8)	96.6 ± 18.0	1.56 (1.12, 2.18)	0.008	

*^a^Data were presented as OR (95% CI) for prevalence of diabetes when plasma copper concentration ≥109.4 compared with those with plasma copper concentration <109.4 μg/dL (reference) estimated by using multivariable logistic regression models. Adjusted confounders included age, sex, current smoking, current alcohol drinking, BMI, family history of diabetes, SBP, DBP, tHcy, TC, and TG*.

*BMI, body mass index; DBP, diastolic blood pressure; SBP, systolic blood pressure; TC, total cholesterol; TG, triglycerides; tHcy, total homocysteine; HDL-C, high-density lipoprotein cholesterol*.

### Restricted Cubic Spline Analysis

**Figure 2:**
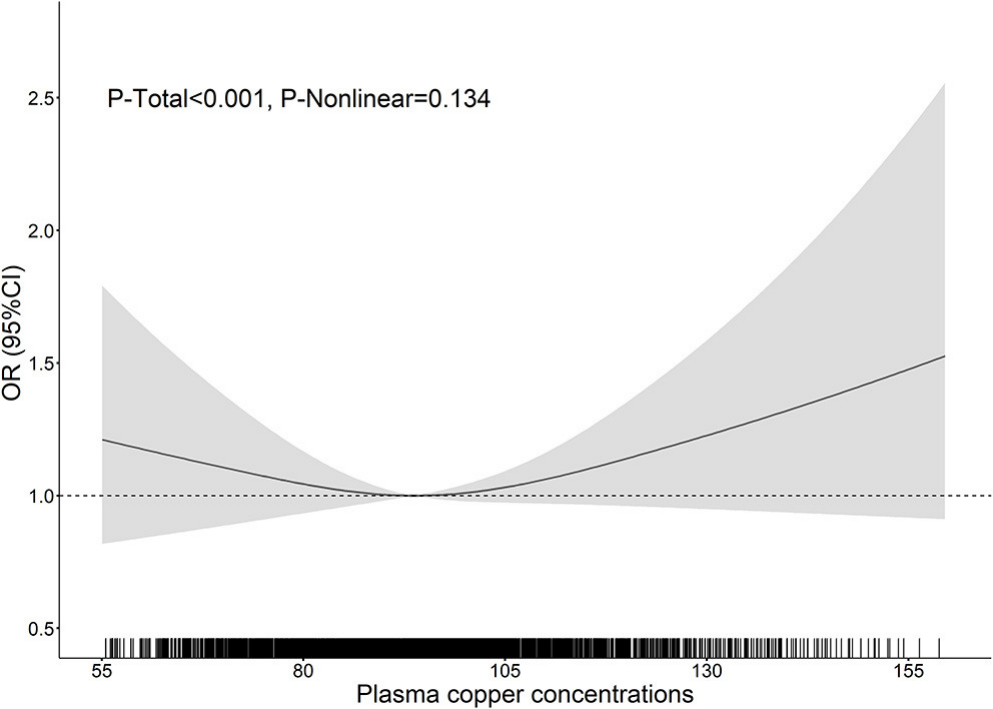
The association between plasma copper concentrations and prevalence of diabetes in 2,579 Chinese adults with hypertension. The association between plasma copper concentrations and the prevalence of diabetes was depicted using restricted cubic spline with three knots (10th, 50th and 90th). Black solid lines were estimated OR of diabetes risk associated with plasma copper concentrations. Gray shade area showed corresponding 95% CI. Dotted lines located at OR as 1 indicating no association. The rugs showed fraction of participants with different plasma copper concentrations ranging from 55 to 160 μg/dL. The OR of diabetes prevalence associated with plasma copper concentrations was adjusted for age, sex, current smoking, current alcohol drinking, BMI, family history of diabetes, SBP, DBP, tHcy, TC and TG. BMI, body mass index; DBP, diastolic blood pressure; SBP, systolic blood pressure; TC, total cholesterol; TG, triglycerides; tHcy, total homocysteine.

The results of RCS analysis showed no significant non-linear relationship between plasma copper concentration and prevalence of diabetes (*P-overall* < 0.001, *P-non-linear* = 0.134; Figure 2).

## Discussion

Trace elements are getting more and more attention in relation to the prevalence of diabetes, including copper (7, 8). Excessive plasma copper concentration has been reported to mediate the formation of damaging ROS, which is associated with insulin resistance (11, 12). Adults with hypertension have elevated serum copper concentrations (27) and their risk for new-onset diabetes is over twice higher compared to healthy normotensive adults. However, the association between plasma copper concentration and prevalence of diabetes in adults with hypertension is unclear. To the best of our knowledge, the current study filled this research gap *via* assessing the association between plasma copper concentration and diabetes in Chinese adults with hypertension. The finding of this study showed that plasma copper concentration was positively associated with prevalence of diabetes in hypertensive adults with serum HDL-C concentration ≥1.2 mmol/L.

In the current study, we found significantly higher prevalence of diabetes in participants with plasma copper concentration ≥109.4 μg/dL compared with those with plasma copper concentration <109.4 μg/dL in Chinese adults with hypertension. Similar to our results, a meta-analysis among the general population including 1,079 diabetic cases and 561 healthy controls from 15 eligible studies has reported significantly higher plasma copper levels in the diabetic cases than in the healthy controls (32). A case-control study has reported that the OR (95% CI) of type 2 diabetes in the highest tertile of plasma copper concentration was 4.21 (3.20, 5.55) compared with the lowest tertile in Chinese Han adults (18). In addition, several case-control studies have also identified a positive association between plasma or serum copper concentration and type 2 diabetes in Chinese adults (17, 19, 33). Conversely, other case-control studies have reported null association between plasma copper concentration and diabetes (22, 23), or no significant differences in plasma copper concentrations between diabetic cases and controls (20, 21). These discordant results may be due to the differences in the concentrations of plasma copper among different study populations, or small sample sizes and failure to adequately adjust for potential confounders, especially biochemical biomarkers, in the latter studies.

Two underlying mechanisms may explain the positive association between higher plasma copper concentration and increased prevalence of diabetes. Copper contributes to increased oxidative stress *via* enhancing the formation of ROS through Haber-Weiss and Fenton-like reactions, and *via* catalytically participating in the generation of hydroxyl radicals by hydrogen abstraction (11). These consequences lead to beta cell dysfunction, decline of insulin secretion and insulin resistance (12, 34). In addition, a previous study has demonstrated that Cu^2+^ ions can stimulate the generation of H_2_O_2_ *via* mediating the process of aggregating human amylin into amyloid fibrils, which may contribute to the progressive degeneration of islet cells in type 2 diabetes (35).

In the current study, we found that HDL-C significantly modified the association between plasma copper concentration and prevalence of diabetes. To be specific, the positive association between plasma copper concentration and prevalence of diabetes was only observed in the strata of participants with serum HDL-C concentration ≥1.2 mmol/L. This result was not surprising because participants with serum HLD-C concentration ≥1.2 mmol/L had significantly higher plasma copper concentrations compared to participants with HDL-C <1.2 mmol/L in the current study, and a positive association between copper and HDL-C concentrations has been reported in previous studies (36). Cu-SOD and H_2_O_2_ system induces lipid peroxidation (13). It has been reported that HDL is more susceptible to copper-induced oxidation than LDL when copper concentration is high (37). Moreover, a recent study has found that HDL associated lipid peroxidation is higher in adults with diabetic in comparison to adults without diabetes (38). Higher plasma copper concentration in participants with serum HDL-C concentration ≥1.2 mmol/L may lead to higher lipid oxidation and subsequent oxidative stress, which may result in higher prevalence of diabetes attributed to beta cell dysfunction, decline of insulin secretion (11, 34) and progressive degeneration of islet cells (35). Assessment of HDL associated lipid peroxidation is required to further explain the underlying mechanism.

The current study has several strengths. To the best of our knowledge, this is the first study to explore the association between plasma copper concentration and prevalence of diabetes in Chinese adults with hypertension, and it is the first to find a modification effect of HDL-C on the association between plasma copper concentration and prevalence of diabetes. Compared to previous studies on the relationship between copper and diabetes, a wide range of biochemical biomarkers were adjusted for as potential confounders in our model, in addition to age, sex, BMI and some lifestyle factors, which may contribute to smaller residual confounding. There are also some limitations in our study. Because of the inherent limitations of the cross-sectional study, we cannot establish the causality between elevated levels of plasma copper and higher prevalence of diabetes. Different types of diabetes were not identified in the current study. Therefore, further studies on the association between plasma copper concentration and prevalence of specific types of diabetes are needed. Dietary copper intake was not measured concurrently as a reflection of copper exposure. In addition, biological mechanisms that may underly the positive association between plasma copper concentration and prevalence of diabetes in hypertensive adults with higher HDL-C concentration were speculated rather than measured in the study.

## Conclusion

In conclusion, there was a positive association between plasma copper concentrations and prevalence of diabetes in Chinese hypertension adults with higher HDL-C concentration. Further studies are needed to confirm the causal relationship between copper status and diabetes in Chinese populations with hypertension and explore the underlying mechanisms.

## Data Availability Statement

The datasets of the current study are available from the corresponding author upon reasonable request.

## Ethics Statement

The studies involving human participants were reviewed and approved by the Ethics Committee of Peking University First Hospital, Beijing, China (Ethics Code: 20161231). The patients/participants provided their written informed consent to participate in this study.

## Author Contributions

ZC, XX, XQ, and HM contributed to study conceptualization and methodology. ZZ and NZ contributed to software. ZW, TL, YS, LL, PC, JG, BW, and HZ contributed to investigation of the study. LL, HC, WL, and PW contributed to data curation and validation. XX, XQ, YD, XH, and GT contributed to resources. ZC contributed to formal analysis, project administration, and writing—original draft preparation. HM, XQ, and YY contributed to writing—review and editing. XX contributed to supervision. XX, XH, and GT contributed to funding acquisition. All authors have read and agreed to the published version of the manuscript.

## Funding

The study was supported by the National Key Research and Development Program (2016YFE0205400, 2018ZX09739010, and 2018ZX09301034003), Key R&D Projects, Jiangxi (20203BBGL73173), the National Natural Science Foundation of China (81960074 and 81773534), Project of Jiangxi Provincial Health Commission (202130440), the Department of Science and Technology of Guangdong Province (2020B121202010), the Science and Technology Planning Project of Guangzhou, China (201707020010), the Science, Technology and Innovation Committee of Shenzhen (GJHS20170314114526143 and JSGG20180703155802047), and the Economic, Trade and Information Commission of Shenzhen Municipality (20170505161556110, 20170505160926390, and 201705051617070).

## Conflict of Interest

The authors declare that the research was conducted in the absence of any commercial or financial relationships that could be construed as a potential conflict of interest.

## Publisher's Note

All claims expressed in this article are solely those of the authors and do not necessarily represent those of their affiliated organizations, or those of the publisher, the editors and the reviewers. Any product that may be evaluated in this article, or claim that may be made by its manufacturer, is not guaranteed or endorsed by the publisher.
