# Supplementary material for: The Association Between Plasma Copper Concentration and Prevalence of Diabetes in Chinese Adults With Hypertension
Source: Front Public Health. 2022 Jun 3;10:888219. doi: 10.3389/fpubh.2022.888219 (PMC9210921; doi:10.3389/fpubh.2022.888219)
Supplement: Supplementary file 1 [file Table_1.docx]

***Supplementary Material***

**Supplementary Table 1. Characteristics of Chinese adults with hypertension between selected and excluded participants ^1^.**

| **Variables** | **Sample 1** | | **Sample 2** | |
| --- | --- | --- | --- | --- |
|  | **Excluded** | **Selected** | **Excluded** | **Selected** |
| N | 5346 | 900 | 18071 | 1709 |
| Age, y | 62.1 ± 11.8 | 62.6 ± 12.5 | 60.3 ± 11.73 | 63.4 ± 13.7 |
| Male, n (%) | 3197 (59.8) | 528 (58.7) | 10722 (59.3) | 868 (50.8) |
| BMI, kg/m^2^ | 25.0 ± 3.5 | 24.8 ± 3.8 | 25.4 ± 3.7 | 25.1 ± 3.6 |
| Current smoking, n (%) | 1175 (22.0) | 216 (24.0) | 4090 (22.6) | 294 (17.2) |
| Current alcohol drinking, n (%) | 944 (17.7) | 176 (19.6) | 3399 (18.8) | 285 (16.7) |
| Family history of diabetes, n (%) | 585 (10.9) | 104 (11.6) | 2191 (12.1) | 202 (11.8) |
| Use of antihypertensive drugs, n (%) | 3199 (59.8) | 562 (62.4) | 11733 (64.9) | 1205 (70.5) |
| SBP, mm Hg | 144.0 ± 17.0 | 143.3 ± 16.3 | 144.1 ± 16.4 | 142.6 ± 17.2 |
| DBP, mm Hg | 87.6 ± 10.7 | 87.3 ± 10.8 | 88.4 ± 11.0 | 86.6 ± 11.9 |

^1^ Data are presented as mean ± SD or n (%). Abbreviations: BMI, body mass index; DBP, diastolic blood pressure; SBP, systolic blood pressure.

| **Supplementary Table 2. The association between serum HDL-C concentration and prevalence of diabetes in 2579 Chinese adults with hypertension ^1^.** | | | | | | |
| --- | --- | --- | --- | --- | --- | --- |
| **Serum HDL-C concentration, mmol/L** | **N** | **No. of case (%)** | **Model 1** | | **Model 2** | |
|  |  |  | ***OR* (95% *CI*)** | ***P*** | ***OR* (95% *CI*)** | ***P*** |
| Continuous, per SD | 2579 | 697 (27.0) | 0.74 (0.68, 0.82) | <0.001 | 0.76 (0.68, 0.83) | <0.001 |
| Quintiles |  |  |  |  |  |  |
| Q1 (< 0.996) | 516 | 186 (36.0) | ref |  | ref |  |
| Q2 (0.996 – 1.150) | 498 | 155 (31.1) | 0.80 (0.62,1.04) | 0.097 | 0.78 (0.60, 1.03) | 0.076 |
| Q3 (1.150 – 1.300) | 525 | 138 (26.3) | 0.63 (0.49, 0.82) | 0.001 | 0.63 (0.48, 0.83) | <0.001 |
| Q4 (1.300 – 1.510) | 502 | 112 (22.3) | 0.51 (0.39, 0.67) | <0.001 | 0.50 (0.37, 0.66) | <0.001 |
| Q5 (≥ 1.510) | 538 | 106 (19.7) | 0.44 (0.32, 0.57) | <0.001 | 0.45 (0.33, 0.60) | <0.001 |
| *P for trend* |  |  |  | <0.001 |  | <0.001 |
| ^1^ Data are presented as *OR* (95% *CI*) estimated by using logistic regression models. Model 1 was crude model. Model 2 adjusted for age, sex, current smoking, current alcohol drinking, BMI, family history of diabetes, SBP, DBP and tHcy. Abbreviations: BMI, body mass index; DBP, diastolic blood pressure; SBP, systolic blood pressure; tHcy, total homocysteine. | | | | | | |
